# A Review of Models Used for Investigating Barriers to Healthcare Access in Australia

**DOI:** 10.3390/ijerph17114087

**Published:** 2020-06-08

**Authors:** Nagesh Shukla, Biswajeet Pradhan, Abhirup Dikshit, Subrata Chakraborty, Abdullah M. Alamri

**Affiliations:** 1 Centre for Advanced Modelling and Geospatial Information Systems (CAMGIS), University of Technology Sydney, 2007 NSW, Australia; nagesh.shukla@uts.edu.au (N.S.); abhirup.dikshit@student.uts.edu.au (A.D.); subrata.chakraborty@uts.edu.au (S.C.); 2 Department of Energy and Mineral Resources Engineering, Sejong University, Choongmu-gwan, 209 Neungdong-ro, Gwangjin-gu, Seoul 05006, Korea; 3 Department of Geology & Geophysics, College of Science, King Saud University, P.O. Box 2455, Riyadh 11451, Saudi Arabia; amsamri@ksu.edu.sa

**Keywords:** access barrier, health outcome, model, review

## Abstract

Understanding barriers to healthcare access is a multifaceted challenge, which is often highly diverse depending on location and the prevalent surroundings. The barriers can range from transport accessibility to socio-economic conditions, ethnicity and various patient characteristics. Australia has one of the best healthcare systems in the world; however, there are several concerns surrounding its accessibility, primarily due to the vast geographical area it encompasses. This review study is an attempt to understand the various modeling approaches used by researchers to analyze diverse barriers related to specific disease types and the various areal distributions in the country. In terms of barriers, the most affected people are those living in rural and remote parts, and the situation is even worse for indigenous people. These models have mostly focused on the use of statistical models and spatial modeling. The review reveals that most of the focus has been on cancer-related studies and understanding accessibility among the rural and urban population. Future work should focus on further categorizing the population based on indigeneity, migration status and the use of advanced computational models. This article should not be considered an exhaustive review of every aspect as each section deserves a separate review of its own. However, it highlights all the key points, covered under several facets which can be used by researchers and policymakers to understand the current limitations and the steps that need to be taken to improve health accessibility.

## 1. Introduction

Appropriate and timely access to healthcare is of the utmost importance; if not provided, it can lead to several concerns like missed scheduled appointments, delayed medication, and potential fatality. The barriers to accessibility are varied and are dependent on location, affected disease and patient characteristics. Australia is a vast country with a very diverse population where settlement is spread thinly over vast areas [1]. The country also has an aging population which will require healthcare support in the future. Therefore, understanding the models used to analyze barriers to healthcare access across various diseases is crucial. In terms of geographic patterns, 31% of the population live in rural and remote areas; they have lower usage rates due to the distance–decay relationship. The distance–decay association suggests that people who live farther from healthcare facilities have lower rates of usage, after the adjustment of other factors for need, than those who live closer [2]. Longer travel times to healthcare facilities may also be associated with worse health outcomes for patients [3]. Weinhold and Gurtner [4] in their review article on health services in rural areas found that the rural areas suffer from a limited and inferior quality of health services when compared to urban areas. The delay to healthcare services due to the transportation obstacle is a critical issue for patients suffering from fatal injury or requiring check-ups and medication. In such instances, the importance of understanding these transportation barriers is more significant than the access itself. The impediment to transportation can also affect the medication of patients and have long-term detrimental health concerns.

The diversity of Australia is quite rich with 2.3% of the population belonging to the Aboriginal and Torres Strait Islander people, who suffer the most in terms of healthcare accessibility. Rolfe et al. [5] highlighted that indigenous people are the most deprived, irrespective of any health measure. Apart from geographical barriers, factors like socio-economic status and cultural bias also seem to be of significance among indigenous people. Li [6] studied the barriers faced by indigenous people and listed several challenges such as cultural differences, linguistic barriers, and economic aspects. The same study also examined the role of healthcare professionals and suggested several measures which could help to overcome the barriers. McBain-Rigg and Veitch [7] conducted a study on the indigenous people residing in north-west Queensland by performing interviews with the people and with health professionals. The interviews revealed that the focus needs to shift towards building trust and interpersonal relationships between the community and health professionals, not only improving the physical environments.

This paper is organized in six sections, wherein the first section introduces the need for such a review. Section 2 discusses the various sources used to collect the relevant articles and the bibliographic and geographical analysis. Thereafter, Section 3 highlights the definition of accessibility and the spatial techniques used to measure it, along with the Australian healthcare system and the various barriers considered to affect accessibility. Section 4 discusses studies on barriers to accessibility related to various diseases, along with the methodologies used and the results obtained. Consequently, Section 5 discusses the points of strength and highlights the present shortcomings. Finally, Section 6 concludes and summarizes the study.

## 2. Data Collection

**Figure 1:**
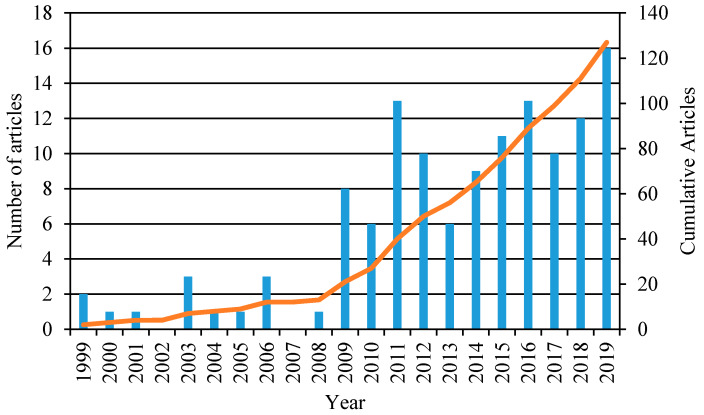
Analysis of the literature database from 1999–November 2019. The left Y axis represents the number of articles per year and the right Y axis depicts the cumulative number of articles for the entire period.

The primary objective behind this review article was to understand the models being studied for barriers related to healthcare access. For this, a bibliographic search was conducted on PubMed and Web of Science for peer-reviewed studies written in English and conducted only in the Australian context. The search was based on various forms of the keywords “Barrier *”, “Model *” and “Health Outcome”. Medical Subject Heading (MESH) terms included health services accessibility, barrier, and health outcome. After conducting the relevant searches and removing duplicates, a total of 127 papers were selected. The distribution of the articles by year is depicted in Figure 1.

The articles were published in 73 different journals, with the highest numbers of publications in Medical Journal of Australia (10%), BMC Health Services Research (5%), Rural and Remote Health (4%), Applied Geography (5%), Australian and New Zealand Journal of Public Health (3%), Australian Journal of Rural Health (4%), Cancer Epidemiology (3%) and International Journal of Health Geographics (3%).

**Figure 2:**
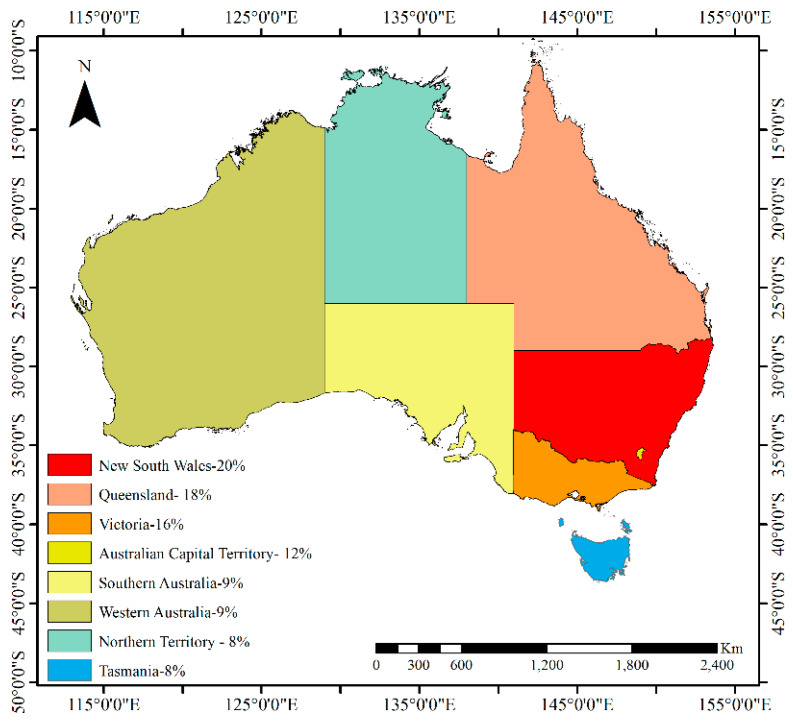
Number of studies conducted across various states of Australia.

We also analyzed the states where the studies were conducted. The results reveal that 20% of the studies were conducted on a national level. The studies conducted in specific states are illustrated in Figure 2. The analysis was performed based on the state where the study was conducted irrespective of covering a small part of the state.

## 3. Definition of Accessibility

Healthcare access was classified into 5 categories in [8], which are: (a) availability, (b) accessibility/proximity, (c) affordability, (d) acceptability and (e) accommodation. The first two (availability, accessibility) can be considered spatial whereas the remaining factors are non-spatial [9]. Geographic Information Systems (GIS) is considered a powerful tool to integrate both spatial and non-spatial factors [10]. However, most studies analyzed hindrance to access in the spatial context irrespective of disease type.

The studies focusing on spatial accessibility were analyzed using three different techniques: (i) distance/time to nearby services, (ii) gravity models and (iii) population versus provider services: doctor-population ratio (DPR) or bed-population ratio (BPR) [11,12]. The first approach is a simple technique wherein the distance between population residence and service provider (proximity) is determined without considering the availability aspect of spatial accessibility. The determination of accessibility was usually carried out by determining travel time. However, some studies used the line-of-sight measure, in which distances were used as a measure of access [13,14,15,16]. This concept can be used in some scenarios as access to cars is one of the highest in the world for people residing in urban areas of Australia, due to a highly developed road network [13]. The second approach considers both aspects (availability and proximity); however, the limitation of using gravity models is a challenge for the determination of the distance–decay function [9,17]. The population versus provider services approach uses a classification of the population and health services within a defined region instead of the spatial movement as used in the other two approaches. The determination of the ratios is easy to compute, as the data for both the population and the health centers are usually available. The use of such an approach involves two assumptions: (a) that the population is expected to use health services within the defined region, and (b) that the proximity aspect is negligible within the region [11]. The significant difference lies in the selection of defined regions. As the name suggests, the floating catchment area (FCA) method uses floating areas or “windows” instead of defined regions, the size of which is determined by the availability of the required services within a region. The use of FCA leads to the major challenge of not considering the demand aspect with respect to supply–demand concerns. This challenge was addressed by [18], which introduced the spatial decomposition method, and this approach was then used by [17], which introduced the two-step FCA or 2SFCA method. The 2SFCA method is performed in two steps, first calculating the size of the population within the catchment area, and then determining the available services in the catchment area.

Evaluation of the accessibility of healthcare is usually conducted using GIS techniques, which measure the travel distance and time required for using public or private transportation systems. The studies can be categorized as revealed accessibility or potential accessibility [3]. Revealed accessibility is the actual time taken to reach health centers, whereas potential accessibility analyses the potential to access healthcare determined using either gravity models or specialized gravity models like the 2SFCA method.

After collecting and determining the relevant health barriers, statistical models were applied to analyze the association of the factors with survivability along with the interrelationship of the barriers. The present review looks at the models used to understand the barriers to healthcare access for various diseases in Australia. The aim is also to analyze survivability or outcomes in relation to the barriers. The review was conducted based on several categories including disease, study area, models used, number of patients, rural vs. urban, consideration for indigenous people, and the dataset (source and time period) used.

### 3.1. Australian Healthcare System

The Australian healthcare system is considered a hybrid model where people can purchase private insurance coverage along with the public insurance they already receive, making both public and private hospitals available [19]. The vast geographical area of the country, varied residential locations and their uneven distribution of population, network of roads and traffic conditions and the allocation of hospital resources lead to an imbalance of health service access for the people [12]. In terms of practicing physicians, Australia has 3.39 per 1000 people, which is one of the highest in the world [19]. It also spends the most on healthcare among the Organization of Economic Cooperation and Development (OECD) countries, which are a consortium of 34 countries dedicated towards developing policies for various social and economic challenges [19]. A detailed understanding of the Australian healthcare system can be found in [20].

It has been well established that following illness, health outcomes can get worse upon traveling a greater distance to health centers. Similar bias is often visible among residents living in rural areas as compared to urban areas. The variation between survivability among rural and urban residents for various health cases has been analyzed by several studies [21,22,23]. The rural population suffers from higher fertility and perinatal mortality rates compared to the urban population. The chance of health cases (e.g., diabetes, high cholesterol, cancer, heart disease) is higher than in the urban population, which lowers their life expectancy by 4 years. The National Rural Health Alliance found that the barriers dividing remote areas from major cities are enormous: for example, in the case of remote/very remote areas, over 58% of people reported not having a specialist nearby as compared to only 6% in the case of major cities. Such startling differences are also present across different disease types and health visits.

### 3.2. Factors Considered/Barriers

**Figure 3:**
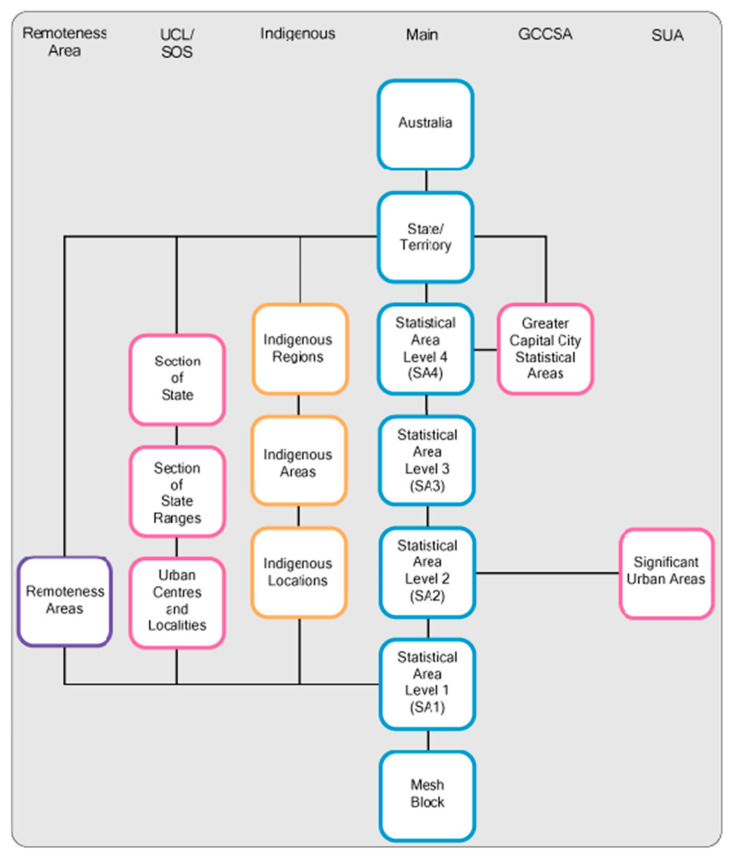
Australian Statistical Geography Standard: Structure (Source: ABS).

The geographical classification of the country is based on the Australian Statistical Geography Classification (ASGC) framework provided by the Australian Bureau of Statistics (ABS) (Figure 3). This classification was initiated in 2011; prior to it the Australian Statistical Geography Standard (ASGS) classification was used. The studies conducted determined the geographical location of their respective study region based on census classification, which has been modified over the years. The population can be based on either place of enumeration (based on the location on census night) or place of usual residence (based on the location where they usually live). The studies relating to healthcare access were conducted based on place of usual residence. Before 2001, the census was based on Statistical Local Area (SLA), which was changed to Collection District (CD) level in the next census. For the 2011 census, the Australian Statistical Geography Standard (ASGS) was used, in which the data were available at Statistical Area 1 (SA1) level, which could be aggregated to higher spatial scales of geography.

**Figure 4:**
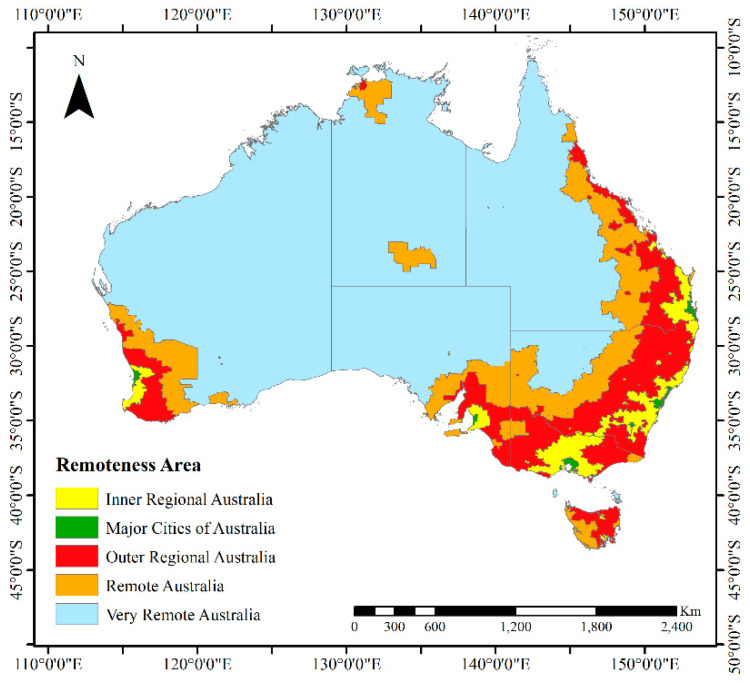
Remoteness map of Australia, 2016 (Source: ABS).

The remoteness of a place can be categorized into one of five classifications: major cities, inner regional, outer regional, remote and very remote [4]. Remoteness has been defined based on the ASGC-RA (Remoteness Area) classification (Figure 4). This classification determines the physical distance of a location and allows the quantitative comparison between metropolitan and rural regions. To compute ASGC-RA, the Accessibility/Remoteness Index of Australia (ARIA+) score is determined. This is an index of remoteness with values ranging from zero (high accessibility) to 15 (high remoteness) based upon the physical distance of a location from the nearest urban center according to census data on population size [24].

The other critical factor while determining accessibility is socio-economic status (SES), which is based on the Socio-Economic Index for Areas (SEIFA) developed by the Australian Bureau of Statistics (ABS) and is a set of four indexes: the Index of Relative Socio-Economic Disadvantage (IRSD); the Index of Relative Socio-Economic Advantage and Disadvantage (IRSAD); the Index of Education and Occupation (IEO); and the Index of Economic Resources (IER). The SEIFA comprises five categories, which are: most disadvantage; above average disadvantage; average disadvantage; below average disadvantage; and least disadvantage [13]. Generally, a socio-economic index is assigned using area-based measurement, which tends to be biased and often inaccurate. This was highlighted by [25] which used individual-based demographic data and compared survival disparity when considering Local Government Area (LGA) and CD classification in the New South Wales region. The results highlight the underestimation of survival disparity with little variation when Relative Excess Risk (RER) is calculated. Factors like patient characteristics including smoking, employment, ethnicity, disability, indigeneity, stigma and discrimination have also been explored by researchers under various circumstances [26,27].

The COVID19 pandemic has revealed new barriers and challenges for healthcare workers and patients affected by it. This has caused patients with several necessary and critical health conditions to prematurely die in several OECD countries. Among the OECD countries, Australia has conducted a commendable job in addressing the barriers for healthcare professionals. Although the situation is still unfolding, a few research articles and news reports are attempting to understand the gravity of the situation. Some have reflected on the emotional state of healthcare professionals [28], while others have suggested the importance of linguistic and communication barriers. In the Australian context, Lakhani [29] a conducted spatial analysis to understand the most vulnerable populations in the Melbourne region depending on their characteristics.

Finally, the survivability of patients is determined by utilizing either the overall survival or relative survival measures. Overall survival is defined as an estimate of survival from the initiation of either the diagnosis or medication, whereas relative survival is defined as an estimate of net survival which measures the deaths specifically associated with cancer diagnoses [22]. Such risks are also dependent on SES. Therefore, relative excess risk (RER) has been defined; this is the ratio of excess risk of death in a particular SES quintile compared to that of the reference (least disadvantaged) SES group, controlling the other factors.

## 4. Results

**Figure 5:**
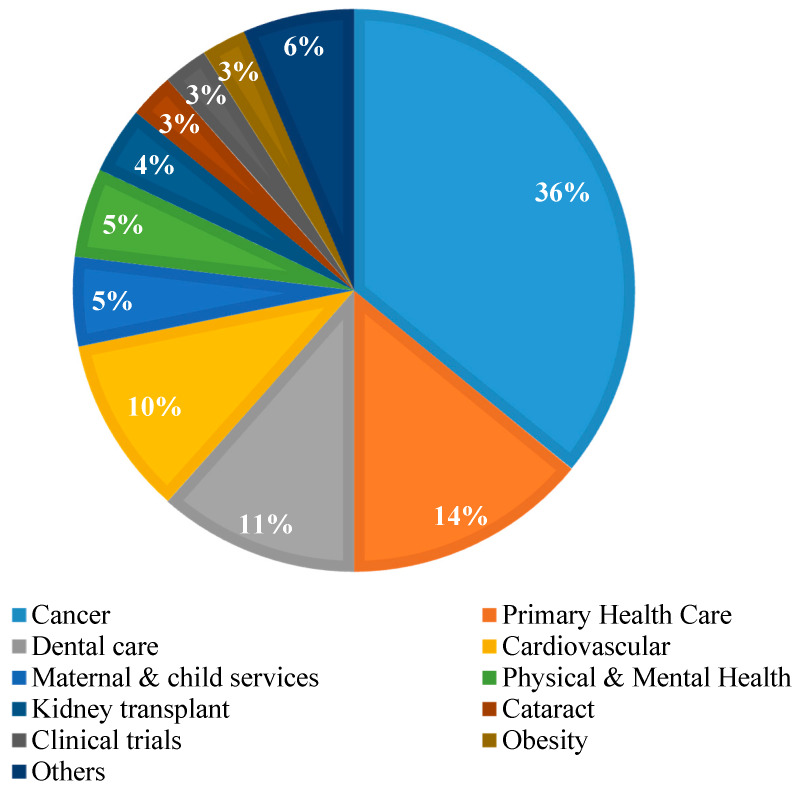
Percentage of studies conducted for various disease types (others include diabetes, disability, frailty, hepatitis C and strokes, each contributing equally).

In terms of diseases, numerous studies have been conducted for various types, of which the greatest number have been performed for cancer (35%), followed by primary health care (14%), dental care (11) and cardiovascular conditions (10%). Figure 5 depicts the studies conducted for various diseases.

Studies focused on understanding the inequalities in healthcare access based on various traits like location (rural, urban), origin (indigenous, nonindigenous), and access to health services. Among the various regions, most studies were performed in Queensland (38%), followed by New South Wales (34%), Victoria (14%) and the entire country (14%).

### 4.1. Cancer

Cancer is the most significant global public health problem and a leading cause of death and illness in the world in the 21st century, including Australia [30]. Breast cancer is estimated to have been the most commonly diagnosed cancer in 2019, followed by prostate cancer. The distribution of the studies related to cancer types has also been varied with most studies being conducted on colorectal cancer followed by breast, prostate and lung cancer. Generally, the studies conducted form a framework in which barriers were analyzed independently as well as in terms of their interrelationship and their relationship with health outcomes. General accessibility factors like age, sex, patient characteristics and disease stage (incidence, various cancer stages) were collected from the respective state’s Cancer Registry. The distance to the health facility was determined by geocoding the distance of all the facilities to the centroid of each SLA or to the address of the patient if available.

There seems to be a set framework when studying barriers to cancer care that considers various geographic and demographic parameters, thereby determining the survival rate. The remoteness index (ARIA+) and socio-economic index are considered when determining the effects on patient survival.

The models used to determine survivability included the Poisson regression model [31] and the Cox proportional hazards model [32]. Survivability can be expressed in either a spatial [33] or a temporal context [34]. Yu et al. [31] used the Poisson regression model to determine survivability by analyzing residential location in diagnoses of colorectal cancer. However, Frowen et al. [35] investigated the impact of pre-treatment factors including demographic parameters. Baade et al. [36] determined the survival rate among colorectal cancer patients residing in Queensland. The study introduced a multilevel approach to assess area-level variation in colorectal cancer survival due to causative factors (disease stage, comorbidity, patient characteristics and healthcare access) and analyze their individual contribution to survival. Baade et al. [32] analyzed the relation between distances to radiotherapy facilities and survival outcomes for rectal cancer patients in Queensland using the Cox proportional hazards regression model. The results revealed that survival rate is low in areas of socio-economic disadvantage, remoteness and greater distance to radiotherapy facilities.

Hsieh et al. [37] quantified the additional barriers that impacted treatment among women in Queensland diagnosed with breast cancer. A Bayesian spatial modeling approach was used to analyze the spatial inequalities of utilizing adjuvant therapy and found that socio-economic aspects did not play a significant role. However, the choice of therapy (radiotherapy, chemotherapy, hormonal therapy) was dependent on the age of the patient. Coory et al. [38] studied the disparity in cancer-related deaths among people residing in regional and remote areas for a period of 10 years (2001–2010). They used an arithmetic methodology wherein the number of deaths precluded in Australia and excess cancer deaths in regional areas were computed. The results revealed a slight improvement in curtailing the disadvantage of such areas, with a death rate lower than metropolitan areas.

An interesting study was conducted in [39], which introduced a new parameter, “country of birth”, along with socio-economic status, remoteness and ethnicity among patients diagnosed with cancer in the New South Wales region. A logistic regression model was used to analyze the relation between variables and the distant summary stage. The results revealed that people born outside of Australia were more likely to be diagnosed, with socio-economic status also playing a significant role.

Mahmud et al. [30] used multivariate analysis to analyze the trends associated with cancer incidence, hospitalization, and fatality for several barriers. The study was conducted for the period 1982–2014 and the results revealed that socio-economic and geographical access play a significant role in patient outcome. Even though there was improvement over the time period, significant improvements need to be made to improve the lifespan of people residing in regional areas.

### 4.2. Primary Health Care

Access to primary health care (PHC) via general practitioners (GP) is critical as a key to improving health outcomes, with more than 80% of people visiting at least once every year [40,41]. Access is quite varied among people residing in rural and urban areas and therefore the focus has been more on understanding access to PHC in rural areas. It has been proven and accepted that with an increase in distance to health centers the utilization of such centers becomes less [42,43]. Studies have primarily focused more on the spatial context.

The 2SFCA method has been heavily used to analyze barriers to primary health care services in Australia for both small and large catchment areas. There have been several improvements in the use of the 2SFCA approach studied in [44]. These improvements include the addition of the distance–decay function and the variable distance–decay function. The distance–decay function included the consideration of distance/time when calculating barriers within a catchment area, whereas the inclusion of the variable distance–decay function considers situations in which travel distance is greater according to the health service required. Such a situation is quite evident in rural areas where a patient may need to travel farther for a specific health service requirement as the services are sparsely distributed. This variation was explored in [45] for the Victoria region where the number of health services was limited to 100 with a travel time of up to 60 min. McGrail et al. [40] developed a National Index of Access which contributed towards an improved understanding of spatial accessibility, which helped locate areas with access disadvantages and could be used for proper health planning. Similar studies were conducted [43,46] for five communities in the Victoria and New South Wales regions and the metropolitan Adelaide region, respectively. The results revealed that travel behavior needs to be considered when analyzing accessibility. However, the variation was understood only by categorizing the population into rural and urban, which may not provide accurate results when analyzing a large study area. This was overcome by the same authors [47] when they defined rules for selection of the catchment area with respect to travel time and the number of health services and performed the study for the entire country. The fundamental challenge of using the 2SFCA method is the definition of catchment areas, and researchers have attempted to define new ways with the ability to accurately assess the disparity in access to GPs in rural and urban regions [48].

However, these studies failed to consider the socio-economic status of the population studied. This aspect was explored in [49], which was performed in the inner regional area of New South Wales. The study applied a bivariate analysis to understand the relationship between remoteness and socio-economic status, leading to the construction of a composite score of deprivation. Thereafter, a pairwise correlation matrix between the number of physicians, remoteness and socio-economic status was performed and validated with the health outcomes. The results revealed that socio-economic status plays a significant role compared to remoteness and physician numbers for determining risk per 1000 persons. Schofield et al. [50] utilized six different variables (sex, age, income, remoteness, health status, employment status) to understand GP access, focusing on people with low socio-economic status residing in rural areas. The results indicated that GP services do not depend on the per capita utilization of the services, irrespective of whether they are based in rural or non-rural areas. However, this relation may not be accurate when considering indigenous people. The inclusion of indigenous people in understanding barriers to accessing PHC services was studied in [41], which highlighted the need for also considering indigenous staff as social and cultural biases may exist. Gibson et al. [51] conducted an in-depth study by reviewing articles related to the barriers faced by indigenous people when assessing PHC.

It is evident that primary health care is probably the most basic and frequently visited health service by the population regardless of region, ethnicity, and socio-economic status. Therefore, it is imperative to understand the various barriers faced by every section of society. The focus has primarily been on understanding the association between remoteness and health outcomes. Several other regions are yet to be explored with the focus shifting towards local areas and considering the social and cultural aspects of the population, which would provide an accurate understanding of these access barriers.

### 4.3. Dental Care

The studies involving dental care were more focused on the spatial understanding of access barriers [13,14,15,16,52]. The focus also seemed to be on analyzing the difference between public and private dental clinics, where roughly 80% of the population visit private clinics [52]. Most of the studies used the line-of-sight method to measure distances to dental care instead of determining travel time as they focused on metropolitan regions with a focus on using geospatial tools to identify accessibility [13,16]. The study in [52] focused on private dental clinics in the Western Australia region and found that rural areas were more disadvantaged compared to the metropolitan areas. McGuire et al. [13] conducted a study in Victoria and found that almost three-quarters of the population resided within 10km of a dental clinic. Almado et al. [16] analyzed dental clinic accessibility for eight metropolitan cities of Australia. The analysis revealed that only 33–50% of people were able to avail of dental services depending on various capital city locations. However, an interesting study was conducted in [26] analyzing the barriers faced by people with disabilities residing in Adelaide. The study was analyzed using bivariate and multivariate models and the results revealed that access was poor for people with disabilities living in rural areas compared to people in community settings. The study also found that a significant barrier to accessing dental care is the unwillingness of dentists to treat disabled people. A similar study was conducted in [27,53] for homeless people in Brisbane and identified fear as a barrier among the homeless population.

### 4.4. Physical and Mental Health

Mental health is essential but can be considered as the poorest service in terms of access, especially in rural and remote areas of the country [54]. Taylor et al. [55] studied the state of patients experiencing mental health issues who needed to be transferred to metropolitan health centers. Qualitative analysis was performed through interviews conducted among six patients and 21 medical staff in the Southern Australia region to understand the barriers faced while transferring patients. Fennell et al. [56] conducted a similar study for adults living in rural parts of South Australia and suggested that health professionals needed to be educated about these barriers. They also used evidence-based approaches to understand the concerns faced by patients. Saurman et al. [54] analyzed the Mental Health Emergency Care (MHEC) Rural Access Project implemented in New South Wales ensuring 24 h access to specialists over video conferencing using a concurrent mixed-methods approach. Wohler and Dantas [57] conducted a review of the barriers faced by immigrant and refugee women when accessing mental health services in Australia. The study highlighted that the barriers include factors like religion, self-reliance and resilience, suggesting that measures need to be undertaken to address these concerns. Maas et al. [58] conducted a spatial analysis using autocorrelation indexes and spatial regression to determine patterns of referral for a mental health program in the Western Sydney region. The results revealed that the distribution formed a pattern covering the areas with low socio-economic status.

The factors affecting easy access to mental healthcare programs are varied and efforts need to be made to analyze the barriers at a local scale and implement steps to overcome them. However, the work surveyed clearly shows that indigenous people, remote areas and low-income people are the most affected.

### 4.5. Heart-Related Studies

Cardiovascular disease (CVD) contributes to almost 35% of deaths in the country and is the second most prevalent disease after cancer [59]. This section discusses studies related to cardiovascular diseases and cardiac rehabilitation services. Studies relied on GIS to determine remoteness and accessibility. Bamford et al. [60] developed Cardiac ARIA to quantify the accessibility of cardiac services via the available road networks. The significant difference between ARIA and Cardiac ARIA lies in the selection of a location for accessibility modeling: ARIA uses population location whereas Cardiac ARIA uses the location of the health service. Cardiac ARIA measures travel time to relevant health centers in two categories: (a) acute Cardiac ARIA, which determines the travel time by ambulance in the event of an acute cardiac arrest, and (b) Aftercare Cardia ARIA, which evaluates the travel time by private transport after hospital discharge. Coffee et al. [59] calculated the Cardiac ARIA index for the entire country based on both categories and concluded that the current system provides timely access for the majority of the population.

Cardiac rehabilitation serves as a primary step for preventing CVD and access to it has been a major concern, especially in remote areas [61]. Higgins et al. [62] reported that the percentage of people attending rehabilitation programs after coronary artery bypass graft surgery varied from 37–66% and identified the lack of effective referral protocols as a major factor. They based their study on patients admitted to the Royal Melbourne Hospital, Victoria, and used a logistic regression model to determine patient characteristics as well as visiting the rehabilitation programs. The uneven distribution of cardiovascular services in the country was highlighted in [63], which argued that barriers are not only confined to distance and transport reliability but are multidimensional, involving other socio-economic parameters. Van Gaans et al. [64] developed the spatial model of accessibility, involving both the geographic and the socio-economic factors. The model determined ratings based on the patients who enrolled in the program versus completion rate of the program.

### 4.6. Other Diseases

The other diseases where the relation between barriers and the health outcome was studied included obesity, kidney transplants, diabetes, strokes, and services such as clinical trials and maternity. The number of people who are obese has increased drastically over the last three decades [65]. Remoteness and socio-economic disadvantage have been found to be the most critical factors affecting obesity [66,67]. The relationship between these factors and body mass index among Australian immigrants was studied using statistical analysis in [65].

In terms of wait listing for kidney transplantation, [21] studied the various barriers faced by patients. The study was conducted using univariate and multivariate models and found that access to the waitlist is based on numerous factors like sex, ethnicity and remoteness. The disparity between indigenous people and nonindigenous people in kidney transplant accessibility was studied by [68]. Statistical analysis including the Cox proportional hazards model was used to understand this disparity.

Scott et al. [69] used regression models to analyze the demographic relationship with healthcare service coverage for the hepatitis C virus. The results revealed that despite the cost of the drug being low, more than 50% of the geographical area treated less than 10% of people suffering with the virus. Gilbert et al. [70] conducted a qualitative study to understand the barriers faced by patients when accessing cataract surgery. They found five significant parameters, i.e., travel time, reputation of the health center, surgeon experience, cost and the wait time for surgery. Sabesan et al. [71] analyzed the willingness for clinical trials among rural and regional patients in North Queensland. Using data from 178 patients and statistical analysis, they found that rural patients are more willing compared to the urban patients. Zdenkowski et al. [72] analyzed the barriers faced by patients when enrolling in a clinical trial for cancer medication. The study was performed by conducting interviews among 188 people under various scenarios ranging from variation in travel time, change in oncologist, trial type and increase in cost. Logistic regression was used, and the results revealed that if the cost and the oncologist remained same, the willingness of participants were greater. However, an increase in travel time led to a decrease in participation, whereas there was no difference concerning trial type.

## 5. Discussion

**Figure 6:**
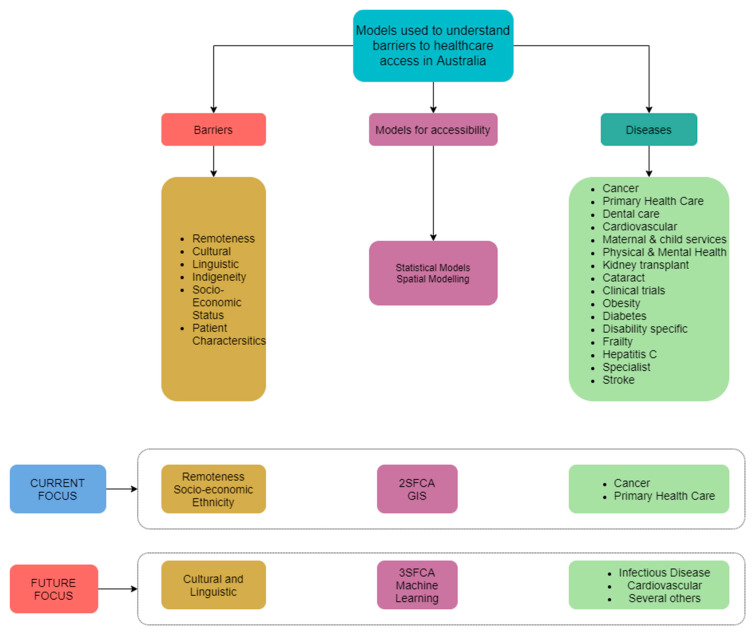
Flowchart of the present study and future directions.

The outcome of this review could be useful for researchers for understanding the various modeling approaches used for understanding barriers to healthcare access in Australia and could also be used in other countries with similar diversity. It provides a broad understanding of the techniques being used, which could serve as a starting point for researchers looking to work in this domain for the first time. The analysis can be useful for identifying some existing shortcomings and the important research questions to be addressed in the future. The findings from the study are illustrated in Figure 6, which depicts three different domains on which the present article has focused, with the various barrier types, the models used to understand the barriers and the diseases for which the study has been conducted. After analyzing all the components of the different domains, we conclude by summarizing the current focus of the research study and providing future directions on which research should focus.

The first gap is the need to focus on other diseases than cancer. Primarily, more research has been conducted towards cancer, which is understandable due to the high number of patients suffering and the rate of fatality. However, more efforts need to be put towards focusing on other major health issues. The second issue is the lack of studies on a finer scale, as most of the studies conducted were either of an entire state or of the whole country. Certain barriers for a specific disease type are pertinent at a local level and their effect on accessibility is also critical. Therefore, emphasis should be on moving towards understanding barriers at a local scale. The COVID19 pandemic has shown the gaps present in the healthcare system in dealing with infectious diseases and our lack of research towards handling barriers for both patients and healthcare workers. Although the Australian health system has considerably performed well compared to other economically developed countries, our understanding of the relevant barriers needs to be comprehensively studied ahead of future infectious disease outbreaks. In general, the main barriers are providing sufficient testing capacities, emotional and physical stress among the health workers and the dispersion of accurate information among the general public.

In understanding various healthcare barriers, accessibility, specifically spatial accessibility, is one specific area where a lot of improvements can be made. The spatial mobility aspect can be considered as the most significant barrier to healthcare access. While the topic has been very well studied in the fields of traffic monitoring and congestion, its application to healthcare studies in Australia has been limited. In terms of the spatial accessibility of health facilities, it can be broadly categorized into two sections: (i) navigation to health centers, which could be proximity to the health center as well as distance or travel time between a certain location and the health center, which would be critical in cases of medical emergencies, (ii) setup of new health facilities, which can be achieved by considering the population demand according to the diseases being suffered from along with considering other factors like affordability, and indigenous status. For both these aspects, the use of GIS integrating with the transport model and the concept of spatio-temporal paths should be encouraged [73]. The effects of spatial accessibility during the pandemic outbreak have revealed some serious gaping holes in the system and its decisionmakers.

### 5.1. Navigation to Health Centers

While the studies in the Australian context focused more on the use of the 2SFCA and other statistical models to calculate distance to health centers, focus should shift towards considering different techniques, e.g., the three-step floating catchment area (3SFCA), which uses distance, proximity and population demand. It could also help in identifying disparities in healthcare access in a regional-level study. Apparicio et al. [74] analyzed the accessibility of health services using various distance and aggregation methods. Such analysis needs to be performed at various spatial scales (national, regional and local) to standardize the basic methodology to be used, which can then be improved in the future. In addition, the input data used for conducting similar studies rely heavily on Google Earth/Maps or OpenStreetMap. Efforts need to be made to use a high spatial dataset [75] which would improve the spatial mobility significant in health scenarios. Such use of a high spatio-temporal dataset would help in identifying the nearest health center along with the shortest route to reach it considering population density [76]. This would immensely support decisionmakers and stakeholders in gaining better access to health centers. The recent work in [77] on determining distance and travel time for Helsinki, Finland using several transportation modes provides a model for deciding the travel mode to be used in cases of medical emergency, clinical check-up and rehabilitation. Such development of a disease-specific travel time dataset, e.g., check-ups for breast and prostate cancer, dental care and GPs, could better aid people in deciding which health facility to go to.

### 5.2. Location of New Health Facilities

It has been well acknowledged that remote areas suffer from an inadequate number of health centers, but the type of health centers for a specific disease type is also quite erratic even in urban areas. Although the specialized field of analyzing the setup of new health centers is a separate entity, we attempted to look at it solely from the different barrier point of view. The lack of facilities can be overcome by setting up new facilities, but the challenges could range from accessibility to cultural difficulties and affordability. The accessibility component can be solved by utilizing the measures mentioned above; however, the other challenges would be detrimental which could be understood by conducting interviews and understanding specific requirements at a community level. The challenge lies in setting up new health centers specific to community-based barriers with the consideration of socio-economic status as well as cultural and regional biases. The steps to set up a new health center could begin with the understanding of broader aspects like accessibility and affordability, and thereafter fill in the gaps of cultural differences with the capacity to upgrade in the future. Another important aspect found while conducting this review was the comparison between rural and urban healthcare accessibility, with a few studies comparing different metropolitan regions. However, comparison between accessibility and health outcomes among the rural regions in a state or across several states was not heavily researched. Such analysis would be interesting to understand which states struggle to provide rural healthcare services and thereafter necessary steps can be taken by the respective state health departments to improve these services. Care must be taken when analyzing the rural regions as patient characteristics like indigeneity, cultural and linguistic barriers would be critical when addressing rural health issues.

## 6. Conclusions

This review paper is an attempt to analyze the models used in understanding barriers to healthcare access and the survivability of the patient across various disease types. Current research practice is lacking in various domains ranging from spatial accessibility techniques to the consideration of patient characteristics and the analysis of different disease types as well as studies concerning only rural/remote areas. Additionally, our understanding of the barriers for infectious disease outbreak is still in infancy and the COVID19 situation would help in determining the various concerns among patients and health workers that should be considered in the future. The study highlighted the key areas on which research has focused: cancer and primary health care-related studies, the 2SFCA method and rural vs. urban health outcomes. The conclusions from the study are as follows:

- It is important to note that the barriers are multifaceted, of which the major ones are geography, ethnicity and socio-economic status. The most deprived section for healthcare access is indigenous people, and this could be even worse if their economic status is poor. The focus needs to shift towards addressing cultural and linguistic barriers, especially for indigenous people. There are also several other barriers which are specific to the disease the patient is suffering from.
- As most studies have focused on a large geographical area, the distance/time determination using the smallest administrative boundary for better accuracy has been missed. The emphasis should be on analyzing at the smallest administrative boundary. The focus has also primarily been on a few diseases only, such as cancer and primary health care, and the location of the study has focused primarily on a few states only. Both aspects need to be improved, the type of disease and the study area.
- The distance/time calculation to health centers is determined spatially using GIS and various geospatial tools. It is encouraging that the available models, such as the 2SFCA method, have been tested very extensively for different regions and have been proved to be performing well. However, new models and techniques like 3SFCA and machine learning need to be attempted for better accuracy. The increase in the availability of data would help in developing machine learning-based tools aimed at identifying key shortcomings and the steps needed to be taken for better healthcare access at both local and regional scales.
